# Supplementary material for: Bayesian refinement of protein structures and ensembles against SAXS data using molecular dynamics
Source: PLoS Comput Biol. 2017 Oct 18;13(10):e1005800. doi: 10.1371/journal.pcbi.1005800 (PMC5662244; doi:10.1371/journal.pcbi.1005800)
Supplement: S1 Archive — (BZ2) [file pcbi.1005800.s011.tar.bz2 › gromacs_4.62_bayesian_saxs_refi/share/html/online/tpr.html]

tpr file format


## tpr file format

|  |  |
| --- | --- |
| Main Table of Contents | **VERSION 4.6** |
| GROMACS homepage | **Sat 19 Jan 2013** |

---

### Description

The tpr file extension stands for portable binary run input file. This file
contains the starting structure of your simulation, the molecular topology
and all the simulation parameters. Because this file is in binary format it
cannot be read with a normal editor. To read a portable binary run input
file type:

```
% gmxdump -s topol.tpr
```

or if you're not such a fast reader:

```
% gmxdump -s topol.tpr | more
```

You can also compare two tpr files using:

```
gmxcheck -s1 top1 -s2 top2 | more
```

---

http://www.gromacs.org
